# A new hybrid algorithm for intelligent detection of sudden decline syndrome of date palm disease

**DOI:** 10.1038/s41598-023-41727-9

**Published:** 2023-09-16

**Authors:** Aurangzeb Magsi, Javed Ahmed Mahar, Abdullah Maitlo, Muneer Ahmad, Mirza Abdur Razzaq, Mohammad Arif Sobhan Bhuiyan, Teh Jia Yew

**Affiliations:** 1 Institute of Computer Science, Shah Abdul Latif University, Khairpur, 66020 Sindh Pakistan; 2 Department of Human and Digital Interface, Woosong University, Daejeon, 34606 Republic of Korea; 3 Electrical and Electronics Engineering, Xiamen University Malaysia, 43900 Sepang, Selangor Malaysia; 4 School of Computing and Data Science, Xiamen University Malaysia, 43900 Sepang, Selangor Malaysia

**Keywords:** Diseases, Engineering, Materials science

## Abstract

Date palm is an important domestic cash crop in most countries. Sudden Decline Syndrome (SDS) causes a huge loss to the crop both in quality and quantity. The literature reports the significance of early detection of disease towards preventive measures to improve the quality of the crop. The number of prevailing detection methods limits to consideration of a certain aspect of disease identification. This study proposes a new hybrid fuzzy fast multi-Otsu K-Means (FFMKO) algorithm integrating the date palm image enhancement, robust thresholding, and optimal clustering for significant disease identification. The algorithm adopts a multi-operator image resizing cost function based on image energy and the dominant color descriptor, the adaptive Fuzzy noise filter, and Otsu image thresholding combined with K-Means clustering enhancements. Besides, we validate the process with histogram equalization and threshold transformation towards enhanced color feature extraction of date palm images. The algorithm authenticates findings on a local dataset of 3293 date palm images and, on a benchmarked data set as well. It achieves an accuracy of 94.175% for successful detection of SDS that outperforms the existing similar algorithms. The impactful findings of this study assure the fast and authentic detection of the disease at an earlier stage to uplift the quality and quantity of the date palm and boost the agriculture-based economy.

## Introduction

Agriculture is the backbone of many countries whose economy relies on agriculture for the total Gross Domestic Product. The agriculture sector of Pakistan involves 42.3% of its total labor force^1^. Pakistan is one of the largest date-producer countries around the globe. In Pakistan, about 93.3 thousand hectares of land are used for the cultivation of Date palms. The maximum quantity of Date fruit in Pakistan is produced in Sindh province^2^. Furthermore, from the perspective of diet, the date fruit is full of nutrients and provides numerous benefits to maintain potential health^3^.

Several diseases and environmental changes affect the agriculture sector. Similarly, some major diseases like Bayoud disease, Black Scorch, SDS, Brown Leaf Spot, Fruit Rot, and Brittle Leaves disease affect Date Palm trees very badly. These diseases cause huge damage to the quality as well as quantity of Date fruit. In this research work, we work on SDS disease detection as this is the major one that heavily affects the Date Palm trees and is spreading in a treacherous pattern in a particular area. Due to its complex pattern of spreading, this disease is very difficult to control which causes a huge loss in the production of Date fruit. Thus, this disease has become a real threat to Date palm cultivation not only in Sindh but across the globe. In addition to such destruction of the date palms, it also restricts its new cultivation^4^.

Computer-based applications are rapidly paving their way in the field of agriculture. Image processing-based techniques. Advancement in technology has reduced human workload as computers in most fields of life are taking over human intervention and the same is happening in the field of agriculture. Various computer vision-based applications have been developed by scholars for the detection of diseases, recognition, and segmentation tasks. This development ultimately leads to unbiased and almost accurate decision-making regarding disease infection and further evaluation.

Based on the problem nature, different researchers used different techniques such as pattern recognition techniques for the identification of diseases in plants, feature extraction for the recognition of fruit types, edge detection for image classification, and so on^5^. In this research work, we propose a novel hybrid fuzzy fast multi-Otsu K-Means (FFMKO) algorithm for the detection of SDS disease in Date Palm trees which has never been addressed yet by any scholar. The proposed algorithm detects the SDS disease at its different stages with the combination of different image feature extraction techniques and Machine learning-based classifiers. Moreover, implementations of methods like Neural Networks (NN) along with the image processing methods such as features extraction and clustering techniques increase the efficiency of the system in the proper detection of disease while color and texture features of Date palm leaves along with the clustering technique helps in the identification of the stage of SDS disease.

## Literature review

The existing literature reports a number of SDS identification methods based on feature extraction techniques, and classification algorithms. Ramesh^6^ performed the task of classification on a large dataset of plant leaves by using Random Forest Approach. The primary goal of their research was to classify healthy and disease-infected leaves of the plants. The research comprised various phases including features extraction and Histogram of an Oriented Gradient technique (HOG). To train the dataset of a large number of images, the authors used a Machine Learning (ML) algorithm which ultimately helps in the proper detection of plant disease. Agarwal^7^ proposed a model of apple disease detection based on three steps i.e., Image Segmentation, Feature Extraction, and Classification, and achieve 98.38% accuracy with the help of K-Means clustering algorithm and Support Vector Machine (SVM).

Similarly, Moallem^8^ proposed a computer vision-based algorithm for apple grading by using a gray-level co-occurrence matrix and multi-class SVM to extract thirteen features of the image and achieved an overall accuracy of 98.38% with the proposed model. Yogesh^9^ presented his research on soybean plant disease identification on mobile-based captured images by using a feature extraction technique decision support mechanism. In addition, Ashok^10^ proposed a model for mango disease detection based on the feature extraction technique of the sequential forward algorithm which extracts the most relevant features from images. The author used NN with the combination of texture features and achieved 90.26% accurate results. Furthermore, Sa^11^ also presented an approach for fruit recognition from images of Deep NN. The authors claimed that their proposed approach could maintain a fast detection process and perform up to a 0.83 F1 score with a field farm dataset with a low burden of ground truth annotation.

Literature reports a lot of research contributions in the field of Plant as well Fruit disease identification. Dia^12^ proposed a model for disease identification in apple fruit at early stages with the use of an electron microscope. Too^13^ also presented a model of disease detection based on a Deep Convolutional Neural Network (CNN) using VGG16, InceptionV4, ResNets, and DenseNets techniques. Henry^14^ also did research on fruit harvesting following the same approach. Likewise, Garcia^15^ described another method of fruit disease identification using feature extraction techniques to detect the infected part of the fruit and received 75% accurate results. Muresan^16^ introduced a method of fruit recognition from a set of high-quality images with the help of a Deep Learning algorithm and presented the results of the trained network in a numerical form. Barbados^17^ presented an investigated research on the factors affecting the design and effectiveness of Deep NN. The author made a research an image database of 50,000 images and made it freely available for any academic use. Similarly, Kamilaris^18^ surveyed the use of Deep Learning in the field of agriculture, and examine major agricultural problems like disease identification and classification by investigating pre-processing techniques, sources, models, frameworks, and their acquired performances. A comparative analysis by the author shows that deep learning techniques provide high accuracy as compared to other methods in classification. Liu^19^ used CNN based AlexNet method and performed experiments on 13,689 images to detect apple fruit disease and gain overall accuracy of 97.62%. It indicates that deep learning-based models are very efficient^20^.

Using methods of ML, particularly CNN, Yang^21^ presented research work on the discovery of genes in plant resistance and achieved desired results in classification. Following the same method, Saldojevic^22^ presented research on the classification of diseased infected leaves of plants with CNN and identified 13 types of different diseases. Furthermore, Krisnandi^23^ performed the disease identification and classification task on tea leaves with 4727 images and achieved 89.64% accuracy in classification using CNN. Wozniak^24^ came up with another architecture based on the ML technique of NN and performed the detection process of a region of interest (RoI) with the help of a heuristic search algorithm which, after segmentation of input images, received selected segmented images and successfully spotted the area of interest.

Awate^25^ detected the fruit diseases using the Open CV library by extracting features like color, texture, and morphology with the help of the K-means clustering approach for segmentation. The authors used Artificial Neural Networks (ANN) for pattern matching and classification of diseases. In the same way, Dubey^26^ presented an analyzing approach for fruits as well as vegetables by extracting features of an image like color and texture and using the K-Means clustering algorithm for image segmentation. The use of SVM in the research performed the classification task with the average classification error of 1% and 3%. Inamdar^27^ also proposed a model for three plant diseases i.e. Bacterial Blight, Leaf spot, and Leaf Rust identification, and used the K-means clustering technique for image segmentation.

Date Palms around the globe are infected with different diseases. Hence, research in this regard further moved toward the comparison of the diseases identified in different parts of the world. They concluded several reasons for the expanding disease on behalf of the comparison. Magsi^28^ made identification and classification of SDS disease using image feature extraction techniques and the NN approach on a dataset of 1200 Date Palm leaf images and acquired 89.4% accuracy.

## Sudden decline syndrome

SDS is the most desolating disease spread destructively in Date palm trees. It has destroyed a large number of Date palm trees around the globe. Since District Khairpur is also terribly affected by this disease. Due to the complex structure of SDS disease, practical research on it is very difficult to conduct. Though, the aggressive expansion of SDS makes an alarming situation for the farmers of Sindh mainly District Khairpur as SDS disease-infected areas are not allowed for Date Palm cultivation. Initial symptoms of SDS are orange-yellowish type marks at the outer layer of the frond’s midrib, and waves towards the central green frond^29^. The growing property of disease dilates drying which as result turns the frond into a completely pale brown.

The disease can infect Date palms in any season as there is no particular season yet designated regarding this malleable disease. Mature and healthy fruits drop in very large numbers due to the attack of this disease. SDS causes a huge loss to Date palms as it dries fruit in bunches when it attacks at various stages. At its last stage, SDS dries the whole tree and is the cause of its devastation. As the result, the infected tree is not able to produce fruit again. Based on experts’ observations, female trees are more secure than male trees from this disease. SDS infects only such female trees which are quite low in health and grow in an adverse environment.

**Figure 1:**
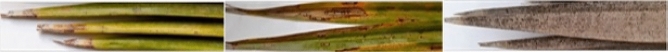
Stage-wise progress of SDS symptoms on front of date palm.

This research work promulgates around the SDS disease due to its severity level. This disease infects stage-wise from the beginning (initial stage) to the ending (last stage), it takes 3 to 4 months to complete the destruction of a tree. We collect the images of Date Palm leaves to carry out the experimentation process and achieve the desired goal. Images for the experimentation were collected from District Khairpur, a city of Sindh Province as it is reported as the most affected city by SDS disease. We distribute the collected Date Palm leaf images into three categories according to their stages. A close snapshot of the SDS progress of symptoms in Date palm is shown in Fig. 1.

## Research methodology

**Figure 2:**
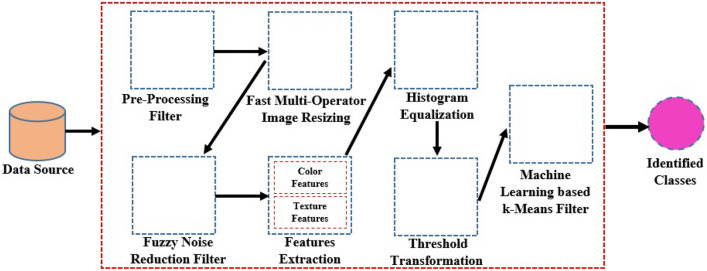
Proposed research methodology.

This study proposes a new hybrid fuzzy fast multi-Otsu K-Means (FFMKO) algorithm integrating the date palm image enhancement, robust thresholding, and optimal clustering for significant disease identification. The algorithm adopts a multi-operator image resizing cost function based on image energy and the dominant color descriptor, the adaptive Fuzzy noise filter, and Otsu image thresholding combined with K-Means clustering enhancements. Besides, we validate the process with histogram equalization and threshold transformation towards enhanced color feature extraction of date palm images. Figure 2 is the depiction of the proposed research methodology.

**Algorithm FFMKO:** Fuzzy Fast Multi-Otsu K-Means

**Inputs:** Problem Set ***P***, Disease Classes ***B***, Preprocess processes ***C***, **FMO** methods

**Outputs:** Final report ***R*** of each problem ***P***

1. Define ***P*** problems from ***S*** problem space where each ${{\varvec{S}}}_{j}$ = $(\text {s}_{1j}$, $\text {s}_{2j}$, $\text {s}_{3j}$), where j $\in$ {1,2,...,N}.
2. Identify disease class ***B*** such that ${{\varvec{B}}}_{q}$ = $(\text {b}_{1}$, $\text {b}_{2}$, $\text {b}_{q}$), where q $\in$ {1, 2, ...,N} based on the available disease types for each problem domain.
3. Prepare/preprocess ***C*** data modules ${{\varvec{C}}}_{p}$ = $(\text {C}_{1}$, $\text {C}_{2}$, $\text {C}_{p}$), where p $\in$ {1, 2, ...,N} depending on data enhancement, normalization, smoothing, augmentation, filtration, etc.
4. For each ***P***j problem, identify **FMO** methods such that each ***P***j charts one or more **FMO** methods.
5. For each **FMO** method, use random data sampling.
6. Validate each **FMO** on ***P*** with required performance parameters ${{\varvec{PP}}}_{a}$ = $(\text {PP}_{1}$, $\text {PP}_{2}$, $\text {PP}_{a}$), where a $\in$ {1, 2, ...,N}.
7. Trace and pick the significant **FMO** method based on performance parameters ***PP***.
8. If step 7 satisfies, and authenticates disease types ${{\varvec{B}}}_{q}$ = $(\text {b}_{1}$, $\text {b}_{2}$, $\text {b}_{q}$), where q $\in$ {1, 2, ...,N}. Produce the final report ${{\varvec{R}}}_{j}$ = $(\text {r}_{1j}$, $\text {r}_{2j}$, $\text {r}_{3j}$), for each ${{\varvec{P}}}_{j}$ = $(\text {p}_{1j}$, $\text {p}_{2j}$, $\text {p}_{3j}$), where j $\in$ {1, 2, ...,N}, otherwise revisit step 4

Let S be the sample space containing random data points, and X be the random variable. We define the mapping as $\text {X}:\text {S} \rightarrow \text {R}$. Where S is the set of the totality of outcomes of the random experiment and X is the function by domain S makes correspondence in terms of real numbers to each outcome. X(t) defines the outcome of the events in the random experiment, here following are the outcomes of the random experiment. $\text {X}(\text {t}_{1})= \text {Disease detection at the first sample}$ for $\text {t}_{1}=1 \le \text {t}_{1} \le \text {J},\,\text {X}(\text {t}_{2})= \text {Disease detection at the second sample}$ for $\text {t}_{2}=\text {J}+1\le \text {t}_{2}\le \text {K},\,\text {X}(\text {t}_{3})= \text {Disease detection at the third sample}$ for $\text {t}_{3}=\text {K}+1\le \text {t}_{3}\le \text {L},\,\text {X}(\text {t}_{4})= \text {Disease detection at the fourth sample}$ for $\text {t}_{4}=\text {L}+1\le \text {t}_{4}\le \text {M}$. $\text {X}(\text {t}_{i}$) Where $\text {t}_{i}$ represents Date leaves with detection at every stage in the random experiment, $\text {t}_{i}\,\in \text {N}$ and J = 335, K = 660, L = 878 and M = 1220. Furthermore, the simple random method is used for the selection of known Date palms Mean with Eq. (1). Where ME is a margin of error, alpha is used to estimate the confidence level, z is used to check the standard score, N is used for the actual size of the leaf, and $\sigma$ is used as input to get the variance of the leaf images. Using equation 3293 leaf images are formed for database development.

1 $$\begin{aligned} N=\left\{ z^2*\sigma ^2* \left[ \frac{N}{N-1} \right] \right\} \Bigg /\left\{ ME^2+\left[ Z^2*\frac{\sigma ^2}{N-1}\right] \right\} \end{aligned}$$

**Figure 3:**
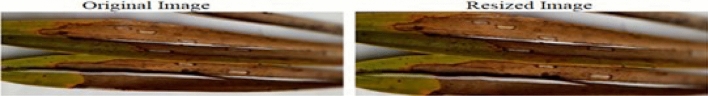
Original and resize images of date palm leaf.

We adopt the image resizing proposed by Dong^30^. With the selected algorithm we use the operating cost method for image resizing using multi-operators resizing techniques like seam craving, image scaling, and cropping technique. The unwanted data in the input image such as extra width and length except the RoI eliminated by using a lower-cost method. During the process of image resizing we formulate the operator cost function with the combination of the image energy and the dominant color descriptor and calculate image parameters as defined in Eq. (2). The detailed process of image resizing is described in^30^ while Fig. 3 is the description of original image along with its resized images.

2 $$\begin{aligned} E(s)=\frac{1}{N_{s}}\sum _{i=1}^{N_{s}} e(S_{i}) + \max _{1\le i\le N_{s}} e(S_{i}) \end{aligned}$$

Where ‘$\text {s}_{i}$’ represents one pixel in the operation field s, $\text {N}_{s}= ||\text {s}||$ is the total number of pixels in s.

During the image acquisition process, we use different digital devices such as Mobile Phone cameras, DSLR cameras, etc. Whereas the images captured with a mobile phone camera bring a lot of noise. Hence, to tackle this issue we prefer a DSLR camera for image acquisition that brings the least noise comparatively. Since the captured images are less noisy yet the noise in images still provides granular effects and spots which are considered part of the image data. For image segmentation and feature extraction, we need a completely noise-free image. We adopted a fuzzy filter to reduce the amount of noise in the input images in this research. The detailed process of the adopted filter is discussed in^31^. Our input image consists of an N × M matrix (a two-dimensional array) of elements where each element of the matrix represents the brightness level and color information. The brightness value of the pixel replaces by a new value d (0–255). However, the noise in the image demonstrates a noise appearance in each pixel (i, j) which has a probability of ‘p’. Let {xi, j} be a distorted image. Then

3 $$\begin{aligned} X_{i,j} = \left\{ d\ with\ probability\ p,\ s_{i,j}\ with\ probability\ (1-p) \right\} \end{aligned}$$

Where ‘$\text {X}_{i,j}$’ represents the random variable, which can have different values based on the assigned probabilities, ‘d’ represents one possible value that ‘$\text {X}_{i,j}$’ can take, ‘$\text {s}_{i,j}$’ is another possible value that $\text {x}_{i,j}$can take.‘$\text {S}_{i,j}$’ is the output brightness of the pixel (i, j). While ‘p’ is the probability associated with the value “d”. It indicates the likelihood of $\text {x}_{i,j}$ taking the value “d”. The value of “p” should be between 0 and 1 and ‘(1 − p)’ represents the complementary probability to “p”. In other words, it’s the probability associated with $\text {x}_{i,j}$ taking the value “$\text {s}_{i,j}$”. Since there are only two possible outcomes, the sum of “p” and “(1 − p)” should equal 1. Equation (3) defines a random variable $\text {x}_{i,j}$ that can take on two values, “d” or “$\text {s}_{i,j}$”. The probability of it being “d” is given by “p,” while the probability of it being “$\text {s}_{i,j}$” is given by “(1 − p)”. Hence, the addition of black and white values of brightness depends on the value of ‘d’ i.e. d = 0 or d = 255.

4 $$\begin{aligned} g(x,y)=f(x,y)+\eta (x,y) \end{aligned}$$

Where f(x, y) is an input image; g(x, y) is a noisy image; $\eta$(x,y) is an additive and independent noise with Gaussian or other distribution of probability density function.

**Figure 4:**
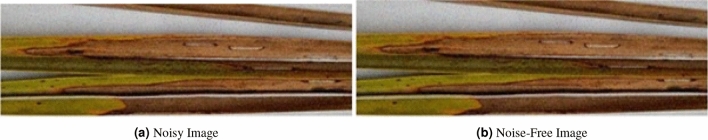
Noisy and noise-free images.

Moreover, we also form an individual class based on fuzzy logic filters to remove more noise from the input image and make it a completely noisy-free image. The class calculates the average value of the central pixel with the reference of neighbor pixel values. This structure makes the perception of boundary on the account of the key object and its color components which then, during the process of distortion, remains undistorted. Filter class calculates the 2-D distance of various color components of the input image which enables the filter to differentiate among the color values of key objects and noise in the image. It is the primary function of this filter class. Figure 4a shows the original image of Date palm leaves with noise and Fig. 4b shows a noise-free image.

After the necessary pre-processing operations, we calculate the image histogram to describe the color components of the input image. Using Fuzzy Color Histogram, we calculate the number of occurrences of each color component in the input image. Given a color space, the color histogram is represented as $\textit{H(I)} = [\textit{h}_{1},\,\textit{h}_{2},\, \textit{h}_{3},\,\ldots ,\textit{h}_{n}]$, where ‘*I*’ represents an input image, h$_{i}$ is the probability a particular pixel in the image then $\textit{h}_{i} = \textit{N}_{i}/\textit{N}$ is the probability of *I* $\sum$ $\text {i}{th}$ color space. The total probability is defined as:

5 $$\begin{aligned} h_{i}=\sum _{j=1}^N \left( P_{i|j} P_j\right) =\frac{1}{N_{s}}\sum _{i=1}^{N} \left( P_{i|j}\right) \end{aligned}$$

**Figure 5:**
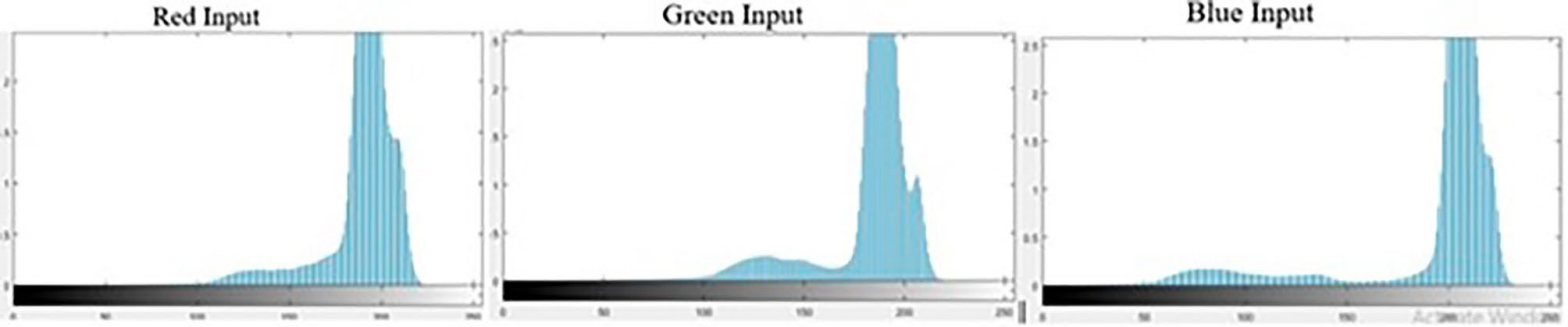
Histogram representation of date palm leaf image.

The probability of the $\textit{j}{th}$ pixel in image *I* is represented as $\textit{P}_{j}$ and is *1/N* whereas $\textit{P}_{i|j}$ represents the conditional probability of the $\textit{j}{th}$ pixel of $\textit{i}{th}$ color space. The graphs of individual color components are generated in this histogram section and stored in a database for future use. Figure 5 is the depiction of generated graphs of individual color components.

In the next step, we use the image thresholding technique to create an image without background for segmentation where the ultimate focus is on the key objectives of the image i.e. RoI and the rest unwanted background data deleted. For image thresholding, we convert the input image into the equivalent grey-scaled image using the Otsu thresholding algorithm which performs automatic conversion of the binary-to-gray level image and then calculated the optimum threshold to separate both classes^32^. By doing so, the combined spread (intra-class variance) is minimal, or equivalently (inter-class variance) is maximal. It calculates the threshold that minimizes the intra-class variance (combined spread) or maximizes the inter-class variance. In other words, it finds the threshold that best separates the two classes. Before applying the Otsu thresholding algorithm, the input image is converted into its grayscale equivalent. Grayscale images represent each pixel’s intensity as a single value ranging from 0 (black) to 255 (white). The conversion is performed to simplify the thresholding process and work with a single channel instead of multiple color channels. The Otsu algorithm determines an optimal threshold value, T, which lies within the grayscale range of 0 to K − 1, where k represents the number of possible gray levels in the image (typically 256 levels for an 8-bit grayscale image). Let a gray-level image f take K possible gray levels 0, 1, 2, ..., k − 1 defines an integer threshold, T, that lies in the gray-scale range of T lies between (0, 1, 2, ..., k − 1). Like the simple comparison process, the thresholding technique is the same hence: each pixel value in f is compared to the threshold, T. Each pixel in the grayscale image, f, is compared to the threshold value, T. If the pixel value is greater than or equal to the threshold, it is classified as foreground, and if it is below the threshold, it is considered part of the background. The value of the corresponding pixel in the output binary image is generated from the above-made comparison. The algorithmic steps use for the image thresholding are:

1. Compute the histogram and probabilities of each intensity level.
2. Set up initial $\omega _{i}$ and $\mu _{i}$. Step through all possible thresholds.
3. Set through all possible thresholds t = 1......... maximum intensity.
  - Update $\omega _{i}$ weight and $\mu _{i}$ (mean).
  - Compute $$\sigma _b^2 (t)$$ (variance)
4. The desired threshold corresponds to the maximum $\sigma _b^2 (t)$.
5. Compute two maxima and two corresponding thresholds $\sigma _b^2 (t)$ is the greater max and is the greater or $\sigma _b^2 (t)$ equal maximum.

6 $$\begin{aligned} desired\,threshold=\frac{threshold_1 + threshold_2}{2} \end{aligned}$$

**Figure 6:**
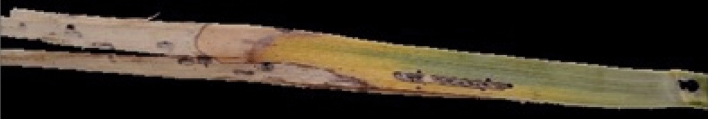
Image background removal.

The process of feature extraction and image segmentation requires a background-free image. Figure 6 is an output image of the Thresholding process while further details about the gray-scale conversion and image thresholding are discussed in^33^.

**Figure 7:**
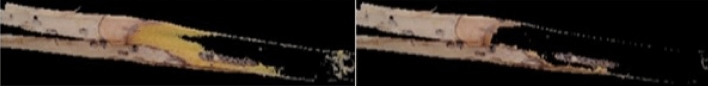
Threshold date palm leaf image.

Right after the background removal using the thresholding technique, the next step is color thresholding where an individual color component of the image such as R.G.B. extracted separately. As the SDS-infected leaf turned pale, yellowish, and brownish. Though except for the green part i.e. healthy part of the leaf the other area represents the diseased infected area. Hence, to calculate the infected part of the leaf, the green-colored area was deleted from the image with the help of the color thresholding technique and exempted the other two color values. Calculations of the other two-color components of the leaf define the infected part along with the description of its size occupied in the leaf. Subsequently, to measure the disease stage, we extract the yellowish part of the image. This extraction of the yellow and dried part of the leaf helps in calculating the infected percentage and that information leads to the decision-making process regarding the treatment and cure of the disease. Figure 7 depicts this part of the process which manifests the division and removal of the color features.

In the feature extraction phase, we extracted the color and texture features of an input image. During the color feature extraction, mainly color brightness and image intensity level were extracted. Values of color features help in the decision-making regarding the disease stage. During the color thresholding process, color features are also extracted but this color feature extraction is somehow different from that happening during thresholding. Here each pixel of the input image was analyzed and extracted RGB color values of each pixel. In Table 1 each box represents a single pixel of the Date palm leaf image consisting of an RGB color component which demonstrates that R, G, and B values of each component vary as per color complexion.

**Table 1:** Color component values of diseased infected part of leaf image.

	Pixel column					
Pixel row	R: 142	R: 144	R: 144	R: 145	R: 145	R: 147
	G: 129	G :129	G: 129	G: 130	G: 130	G: 129
	B: 123	B: 124	B: 124	B: 125	B: 125	B: 125
	R: 131	R: 132	R: 132	R: 132	R: 132	R: 132
	G: 118	G: 119	G: 119	G: 119	G: 119	G: 119
	B: 102	B: 103	B: 103	B: 103	B: 103	B: 103
	R: 133	R: 137	R: 134	R: 133	R: 133	R: 131
	G: 121	G: 123	G: 122	G: 123	G: 121	G: 119
	B: 95	B: 96	B: 96	B: 96	B: 95	B: 94
	R: 140	R: 140	R: 138	R: 138	R: 138	R: 135
	G: 132	G: 135	G: 130	G: 133	G: 130	G: 130
	B: 93	B: 95	B: 93	B: 95	B: 93	B: 92

Irrespective of the difference between primary as well as secondary colors, we extracted and calculated individual color values of each component of the pixel. This step is processed on every pixel of an input image. Separate variables are created for each color component as shown below through which we calculate the ratio of RGB.

$$\begin{aligned} \text {R}{:}\text {x}1,\,\text {G}{:}\text {x}2,\,\text {B}{:}\text {x}3 \end{aligned}$$

The information of each color component is calculated with an average formula which is:

7 $$\begin{aligned} (x1+x2+x3) / n(x) \end{aligned}$$

Where, x1, x2, and x3 variables represent RED, GREEN, and BLUE components of a particular pixel respectively. However, n(x) represents the total number of color components of a selected pixel. The average formula applied on all the selected images of each stage where the notable color ranges vary from stage to stage as shown below:

$$\begin{aligned} \text {Stage-1:}\,151.6863 - 157.2941 \\ \text {Stage-2:}\,158.3271 - 164.9572 \\ \text {Stage-3:}\,165.3928 - 171.3216 \end{aligned}$$

After the extraction of the color feature, generated values are stored in a database using variables. Texture feature extraction is performed as the next step using the Local Binary Pattern (LBP) method. Various Local binary patterns such as Extended Local Binary Pattern (ELBP), Completed Local Binary Pattern (CLBP), Rotation-Invariant Local Binary Pattern (RILBP) and Circular Local Binary Pattern (CLBP) applied to gain the desired results in texture feature extraction but failed to do so. As the state-of-the-art variants of traditional LBP have disadvantages like Increased computational complexity, Higher memory requirements, Sensitivity to noise and Lack of standardized parameters. Hence, based on the requirements a simple and conventional local binary pattern algorithm is implemented in this research work. In texture feature extraction we extract spatial variations of each pixel’s intensity where the input image is first converted into its equivalent gray-scaled image then extracted the values of each pixel of the gray-scaled converted image. On behalf of the extracted pixel values, a three-by-three matrix of pixels of the selected image is formed to apply LBP which helps in the extraction of texture values.
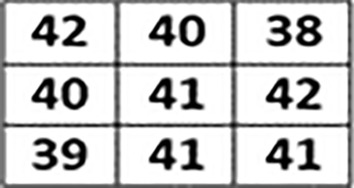


Image texture values calculated using below defined LBP formulae:

8 $$\begin{aligned} f(LBP) = \sum _{n=0}^7 s(i_n - i_c)2^n \end{aligned}$$

Where,

$$\begin{aligned} \text {i}_c= & {} \text {Centre Pixel value }\\ \text {i}_n= & {} \text {Neighbor Pixel value} \end{aligned}$$

Accordingly:
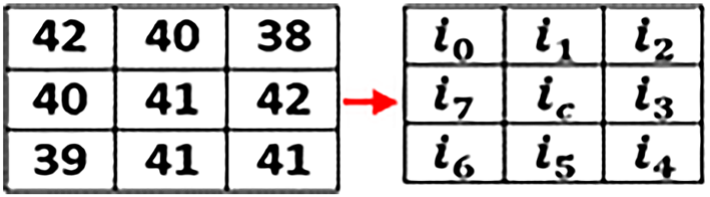


The value of $s(i_n-i_c ) 2^n$ calculated using $s(z) =\left\{ _{0, \ z < \ 0}^{1, \ z\ge \ 0}\right\}$

However, the values from i$_0$ to i$_7$ calculate by:

For, n = 0

9 $$\begin{aligned} s(i_0 - i_c)2^n = s(42-41)2^0= s(1)2^0 = s(1)1 = s(1) \end{aligned}$$

By increasing the number of the neighboring pixels i.e.i$_2$, i$_3$, ...,i$_7$, the values for $s(i_n - i_c)2^n$ for each neighboring pixel are:

$$\begin{aligned} s(i_1-i_c ) 2^n= & {} s(40-41)2^1 = \text {s}(-1)2 = \text {s}(-1)2 = \text {s}(-2) \\ s(i_2-i_c ) 2^n= & {} s(38-41)2^2 = \text {s}(-3)4 = \text {s}(-12)1 = \text {s}(-12) \\ s(i_3-i_c ) 2^n= & {} s(42-41)2^3 = \text {s}(1)8 = \text {s}(1)8 = \text {s}(8) \\ s(i_4-i_c ) 2^n= & {} s(41-41)2^4 = \text {s}(0)16 = \text {s}(0)16 = \text {s}(0) \\ s(i_5-i_c ) 2^n= & {} s(41-41)2^5 = \text {s}(0)32 = \text {s}(0)32 = \text {s}(0) \\ s(i_6-i_c ) 2^n= & {} s(39-41)2^6 = \text {s}(-2)64 = \text {s}(-2)64 = \text {s}(-128) \\ s(i_7-i_c ) 2^n= & {} s(40-41)2^7 = \text {s}(-1)128 = \text {s}(-1)128 = \text {s}(-128) \end{aligned}$$

and as a result, the equivalent conversion matrix is:
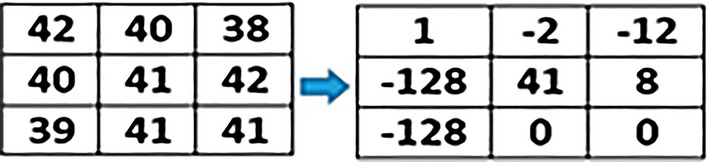


By applying:

10 $$\begin{aligned} s(z) =\left\{ _{0, \ z < \ 0}^{1, \ z\ge \ 0}\right\} , 1 > 00 \end{aligned}$$

We get an equivalent binary matrix for all the neighboring pixels (except the central pixel value i.e. i$_c$).
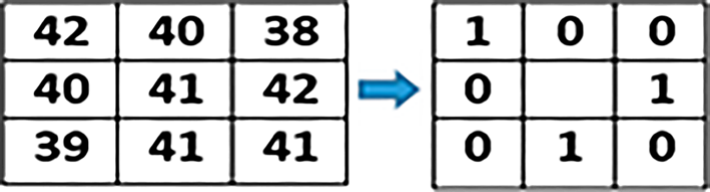


Calculating the decimal numbers of the generated binary matrix results in the generation of a central pixel value for the equivalent matrix.
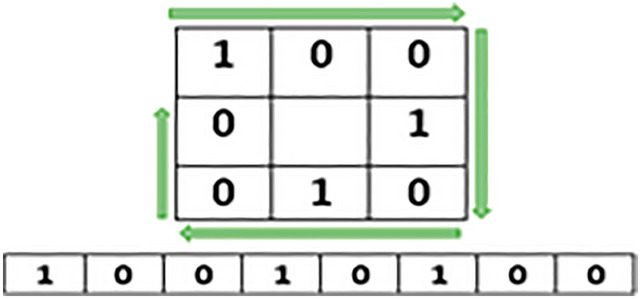


In the next step, all the generated binary numbers are converted into decimal numbers using binary conversion formulae:
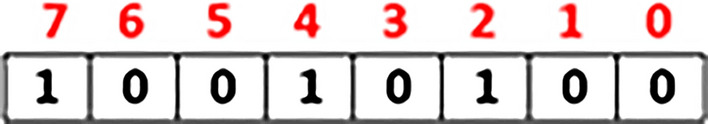

$$\begin{aligned} & = 0\text {x}2^0 + 0x2^1 + 1x2^2 + 0x2^3 + 1x2^4 + 0x2^5 + 0x2^6 + 1x2^7 \\ & = 4 + 16 + 128 \\ & = 148\,\text {(LBP Generated Code)} \end{aligned}$$

Put the generated value of the pixel into the matrix
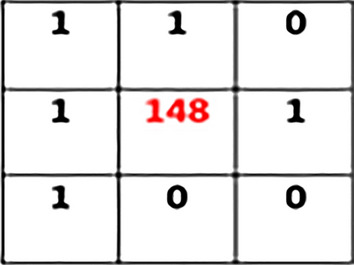


## Results and discussion

The Khairpur District contributes majorly to the production of Date Fruit in Pakistan. With the help of experts from the Botany Department Date palm grower, nearly about 70 date palm fields were visited, each containing approximately 300–1500 date palms. The rapid growth of the SDS disease overspread widely and infected over 7% of the total date palms. Approximately, 3500 infected date palms were identified and the leaf images for experiments were collected manually from the orchards of Khairpur. The collected images of Date Palm leaves were assessed by local field experts and professionals working at Date Palm Research Institute, Shah Abdul Latif University then classify according to the stages of SDS disease accordingly.

For the experimentation process, a total of 3293 images of Date Palm leaves were collected. Images collection assure all the stages of the said disease of SDS i.e. less infected leaf images, images with moderately infected leaves, leaf images with maximum infection, and completely infected leaf images. 3186 images were selected for model training and testing purpose where 75% of the total selected images i.e. 2389 images set for training purposes and 25% of the selected data set i.e. 797 images use for testing purposes. However, 107 images from the total captured images were set as a validation dataset. All the collected images are stored in a database. Table 2 is the complete description of collected images based on the SDS disease stages.

**Table 2:** Information of captured, training and testing images.

Category	Captured images	Training images	Testing images	Validation dataset
Sample-one	535	513	128	23
Sample-two	660	628	157	32
Sample-three	878	854	214	24
Sample-four	1220	1191	298	29
Total	3293	3186	797	107

Experiments require images at the standard level. To fulfill the requirement we capture all the images with a standard camera known as DSLR. To avoid the luminosity issue in images, the image acquisition process performs at various times such as morning, afternoon, and evening. Meeting the international standard of the image of 120,000 lux luminance, 3186 images were selected for experiments. Furthermore, selected images were converted into 300 dpi for better processing.

**Figure 8:**
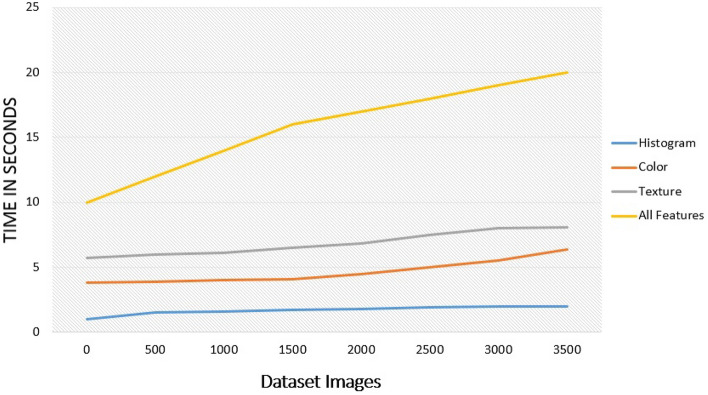
Time taken by features extraction techniques with database images.

Based on the edges achieved in the texture feature, the area of the affected part calculates against the entire area of the leaf. Thus, this feature increases the accuracy with the support of the color feature. Figure 8 presents the graphical representation of time taken by features extraction techniques with dataset images. The histogram technique takes less time and all features together take more time.

Segmentation performs with the help of the K-means clustering algorithm. After the image enhancement by histogram equalization, the K-means clustering algorithm performs the segmentation task on the input image by sub-dividing it into several parts to extract the RoI i.e. diseased infected area of the leaf. The input dataset is divided into clusters in this technique where every cluster is the adaptive representation of changing center. We provided a basic validation metric based on the intra-class and inter-class clusters distances that makes it possible to determine the cluster count automatically. The fundamental process entails creating all segmented images for 2 clusters up to $K_{max}$ clusters, where $K_{max}$ denotes a maximum number limits of clusters which is 4. Then our validity measure is calculated to determine which is the best clustering by finding the minimum value for our measure. An initial value is assigned to start the process. The k-means clustering algorithm calculates the distance between the input value and the center value using the distance value as an input for the nearest center. A vector space is formed for the clustering process with the help of data features which further performs the natural clustering detection process. The objects clustered around the centroids $\mu _i \forall$ i = 1...k and computed by minimizing the following objective

11 $$\begin{aligned} V=\sum _{i=1}^k \ \sum _{x_j\in S_i} (x_j-\mu _i)^2 \end{aligned}$$

Where *k* is the number of clusters i.e. $\textit{S}_i$, i = 1, 2 , . . . , k and $\mu _i$ i is the mean point or centroid of all the points x$_j \in S_i$.

**Figure 9:**
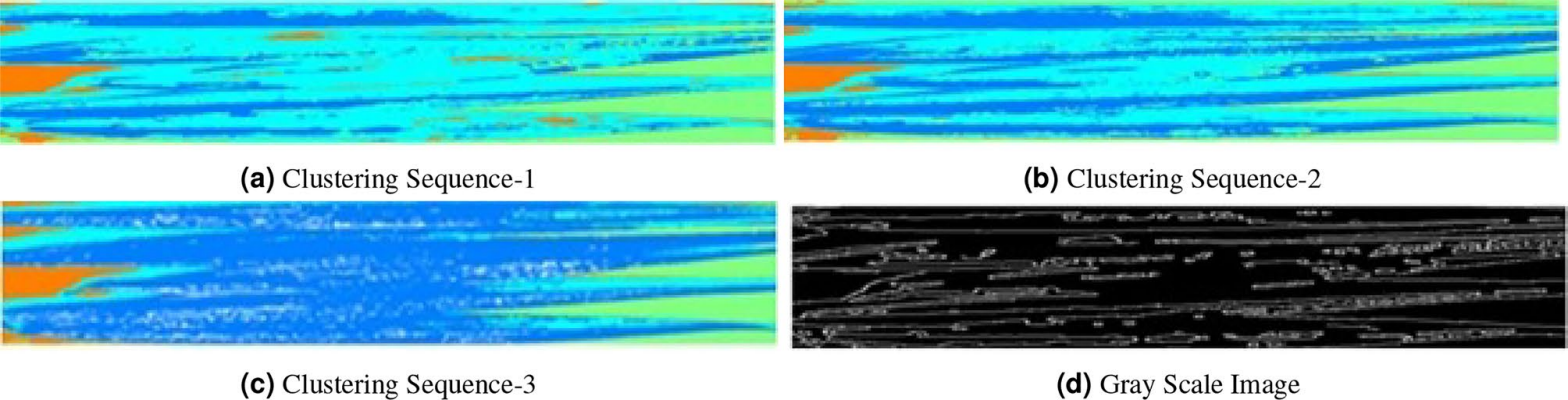
Slightly affected leaves.

**Figure 10:**
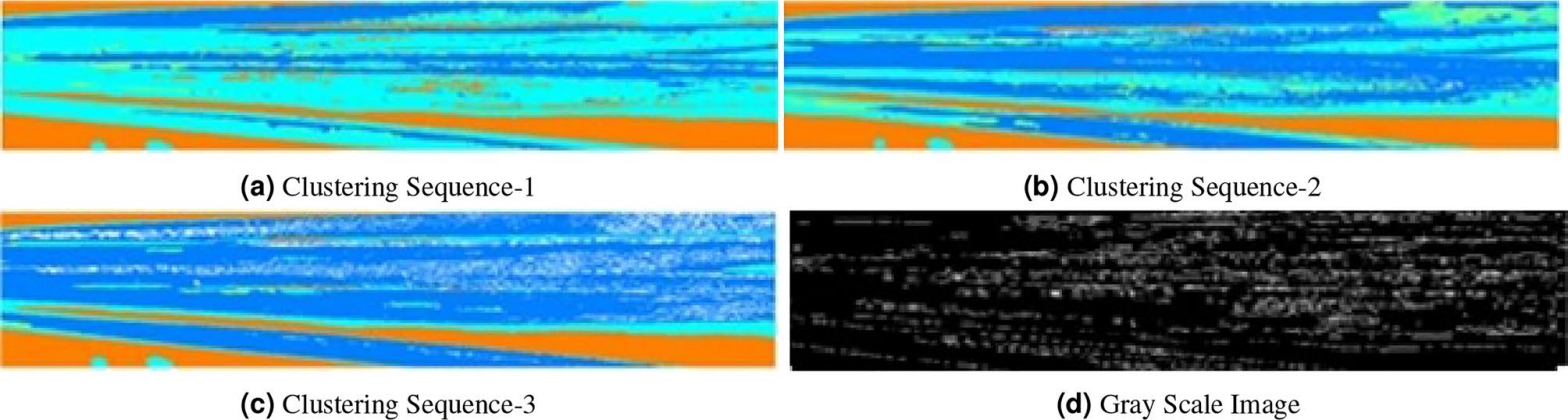
Moderately infected leaves.

**Figure 11:**
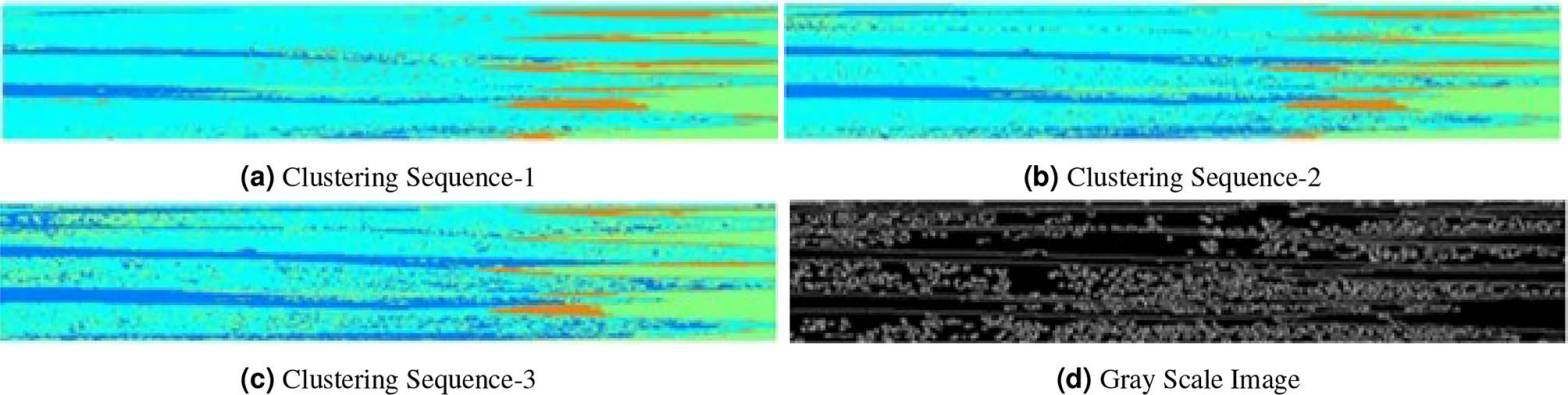
Highly infected leaves.

Most of the researchers obtained desired results from leaf images by implementing the k-means clustering algorithm. Here we process the color image of the Date Palm leaf through a k-means clustering algorithm which performs the image segmentation process and can find a very clear image of the primary object separated from the secondary background. Color as well as the texture of the image are used for the implementation of a clustering algorithm. Throughout the process, various clusters are generated whereas the infected part of the leaf image is selected as an individual cluster. The input image converts into the equivalent gray-scaled image and morphological operations like gradient mask are applied to the gray-scaled image. Figure 9 illustrates a series of clustered images of slightly affected date palm leaves showing that each iteration process makes disease infected part clear. In the original image, it is therefore very difficult to predict the disease level as most of the part of the leaf is looking healthy with the naked eye; however, after each iteration, the implemented algorithm reaches close to the disease and in the fourth part of Fig. 9, the dark blue area of leaf clustered image shows the infected part of the leaf. A gray-scale conversion of the third image is also presented for visualization effects. Moderately infected leaves and highly infected leaves are depicted in Figs. 10 and 11 respectively.

The percentage of the SDS Infected area of a leaf is measured by calculating the total area and the affected area. Following is the equation for calculating the infected area of the Date Palm leaf.

12 $$\begin{aligned} Total\ infected\ area = \frac{Area\ of\ disease\ detected\ in\ image}{Area\ of\ the\ leaf\ image} \times 100 \end{aligned}$$

The collected leaf images were divided into four sample groups as described in Table 1. Experiments were performed on all the samples individually as well as collectively. However, Table 3 presents the accuracy of SDS detection obtained with sample-1 having 128 images.

**Figure 12:**
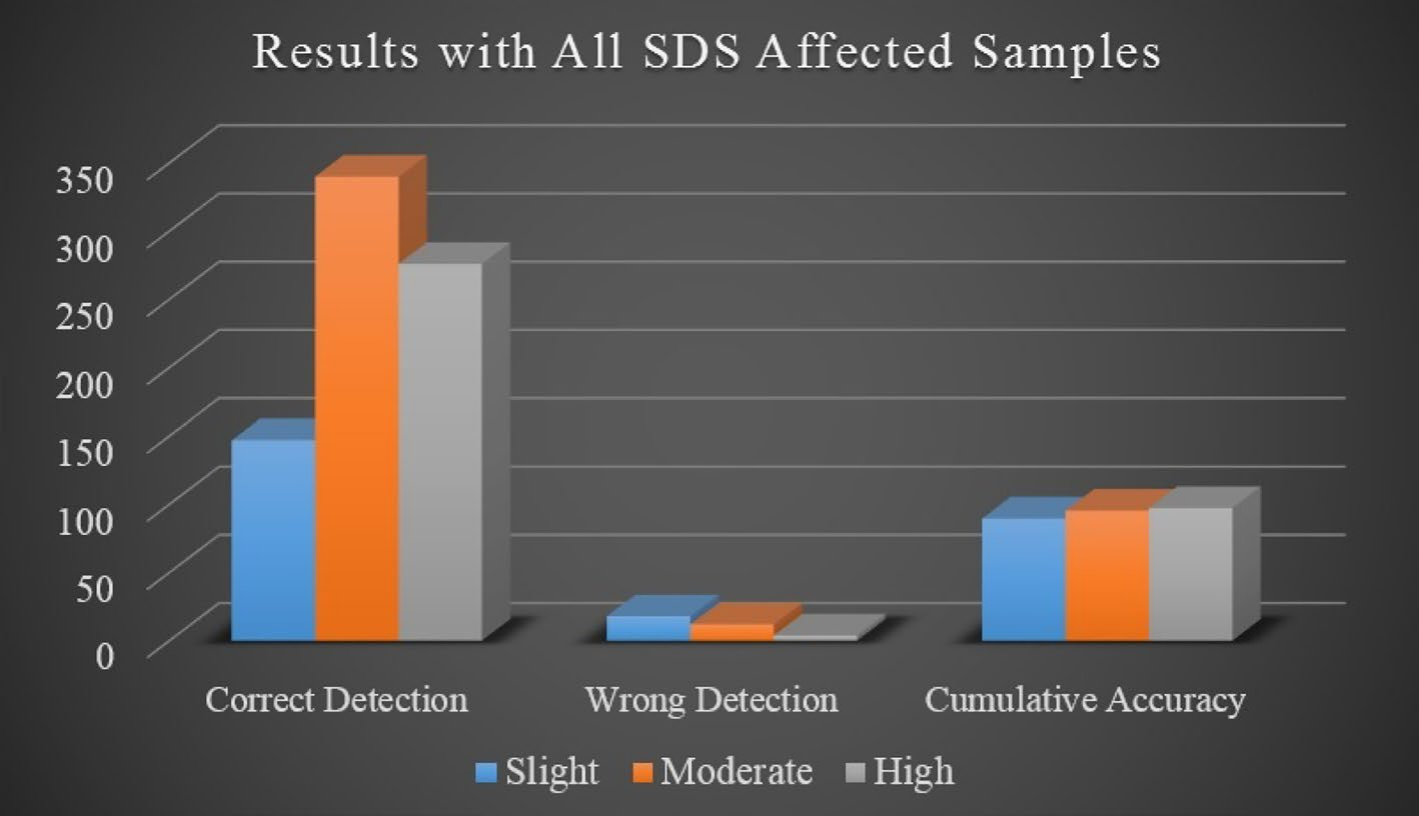
Cumulative performance with all SDS affected samples.

The overall accuracy of SDS disease detection is presented in Table 4 whereas the detection process is described in terms of correctly detected and incorrectly detected leaf images. The experimental performance is represented as detection accuracy which depicts in Fig. 12. The proposed research methodology provides 94.1747% accuracy as the aggregate performance of the system.

**Table 3:** SDS detection accuracy with sample-one.

Infected leaf parts distribution	SDS sample	No. of images	Correct	Wrong	SDS detection accuracy (%)
Slightly affected	1	63	56	07	88.8889
	2	26	25	01	96.1538
	3	18	16	02	88.8889
	4	13	12	01	92.3077
Moderately affected	1	39	36	03	92.3077
	2	48	45	03	93.7500
	3	166	162	04	97.5904
	4	13	12	01	92.3077
Highly affected	1	26	25	01	96.1538
	2	38	37	01	97.3684
	3	30	29	01	96.6667
	4	186	185	01	99.4624
SDS samples		Sample-1	Sample-2	Sample-3	Sample-4
Mean accuracy (%)		92.4501	93.2836	94.382	96.5833

From the obtained results, the performance of the model shows high accuracy in the detection of SDS disease with the sample-4 images which comprise a majority of the highly affected leaf images. On the contrary, it also shows less accuracy with the slightly affected leaf images. It is because of the small number of extracted features and poor performance of k-means clusters. The better performance requires more clear images for experimentation which helps in the extraction of minute features of the input image.

**Table 4:** Overall accuracy of SDS detection with all samples.

SDS disease samples	Infected leaf parts distribution	Average affected area (%)	Total no. of images	Correct	Wrong	Average SDS detection accuracy (%)
All collected samples	Slightly affected	11.2	165	147	18	89.7045
	Moderately affected	48.5	352	340	12	95.4069
	Highly affected	93.7	280	276	04	97.4128
Total			797	763	34	Overall accuracy = 94.1747

## Conclusion

Date palm is a very important domestic cash crop and has very significance in various countries of the world. The disease known as Sudden Decline Syndrome (SDS) significantly reduces the crop’s quality and its yield production ratio. This study proposes a new hybrid fuzzy fast multi-Otsu k-Means (FFMKO) algorithm integrating the date palm image enhancement, robust thresholding, and optimal clustering for significant disease identification. The algorithm adopts a multi-operator image resizing cost function based on image energy and the dominant color descriptor, the adaptive Fuzzy noise filter, and Otsu image thresholding combined with k-Means clustering enhancements. Besides, we validate the process with histogram equalization and threshold transformation towards enhanced color feature extraction of date palm images. We authenticate our findings on a local dataset of 3293 date palm images and, on a benchmarked data set as well. Dataset images divided into four samples i.e. sample-1 consists of less infected images, Sample-2 of moderately infected leaf images; while sample-3 and sample-4 are consisted of highly infected leaf images. Experiments were performed on all the samples individually and collectively as well where the outcomes show that the system achieves 92.45%, 9.28%, 94.38%, and 96.58% accuracy in disease detection and its stage classification with each sample respectively. The algorithm also achieves a cumulative accuracy of 94.175% for the successful detection of SDS outperforms the existing similar algorithms. The impactful findings of this study assure the fast and authentic detection of the disease at an earlier stage to uplift the quality and quantity of the date palm and boost the agriculture-based economy.

## Data Availability

Data will be available upon request from Muneer Ahmad.
